# Evaluation of Pulmonary Artery Acceleration/Ejection Time Ratio (PATET) and the AST-to-Platelet Ratio Index (APRI) in Intrahepatic Cholestasis of Pregnancy

**DOI:** 10.3390/jcm15103632

**Published:** 2026-05-09

**Authors:** Mukremin Ceylan, Abdulmecit Oktem, Ilayda Gercik Arzik, Mucahit Furkan Balci, Zubeyde Emiralioglu Cakir, Hakan Golbasi

**Affiliations:** 1 Department of Perinatology, Health Sciences University, City Hospital of Izmir, Izmir 35540, Turkey; mukreminceylan68@gmail.com (M.C.); abdulmecitoktem72@gmail.com (A.O.); drhkngolbasi@gmail.com (H.G.); 2 Department of Obstetrics and Gynecology, Torbali State Hospital, Izmir 35860, Turkey; mucahitfurkanbalci@gmail.com; 3 Department of Perinatology, Democracy University Buca Seyfi Demirsoy Education and Research Hospital, Izmir 35380, Turkey; zubeydeemiralioglu@hotmail.com

**Keywords:** intrahepatic cholestasis of pregnancy, PATET, PAAT, APRI, fetal Doppler, bile acids

## Abstract

**Objective:** To evaluate fetal pulmonary artery hemodynamics (PAAT, PAET, PATET) in pregnancies complicated by intrahepatic cholestasis of pregnancy (ICP) and to investigate their association with the aspartate aminotransferase-to-platelet ratio index (APRI). **Methods:** In this prospective study, 64 ICP cases and 64 healthy pregnancies are included. Doppler measurements of the fetal main pulmonary artery, umbilical artery (UAPI), and middle cerebral artery (MCAPI) were performed by a single operator, and all biochemical analyses were conducted in the same laboratory. APRI was calculated using the standard formula. **Results:** Doppler evaluation demonstrated significantly higher PAAT, PAET, PATET, UAPI, and MCAPI values in the ICP group (all *p* < 0.05). AST, ALT, and APRI levels were markedly elevated in ICP pregnancies (all *p* < 0.001). No significant correlation was observed between PATET and APRI (*p* = 0.368) or fasting bile acid levels (FBA) (*p* = 0.116), whereas APRI showed a weak positive correlation with FBA (*r* = 0.308; *p* = 0.013). Doppler parameters and APRI values did not differ significantly according to cholestasis severity (10–39/≥40/≥100 μmol/L; all *p* > 0.05). **Conclusions:** In ICP, fetal pulmonary artery Doppler indices (PAAT, PAET, PATET) and fetoplacental Doppler parameters are increased; the elevation in PATET is consistent with lower—rather than higher—fetal pulmonary vascular resistance, potentially reflecting accelerated fetal lung maturation or a hemodynamic adaptation to ICP-related physiological perturbations. Despite elevated APRI levels, these biochemical changes do not parallel fetal hemodynamic indicators. These findings suggest that fetal hemodynamic effects in ICP may be independent of biochemical disease severity. PATET is best conceptualized as a hemodynamic monitoring variable complementing bile acid assessment in fetal surveillance, not as a standalone screening or prognostic tool.

## 1. Introduction

Intrahepatic cholestasis of pregnancy (ICP) is a pregnancy-specific liver disorder that typically manifests in the third trimester and is characterized by pruritus and elevated serum bile acid levels. Although maternal prognosis is generally favorable, it is well established that increased bile acid concentrations may exert serious adverse effects on the fetus [1]. The prevalence of ICP varies according to geographic and ethnic factors, with higher rates reported in South America and Scandinavian countries [2]. Hormonal changes, impaired hepatic bile transport mechanisms, and genetic susceptibility are thought to contribute to the pathophysiology of the disease [3].

Beyond maternal symptoms, ICP is associated with significant fetal complications. Bile acids can cross the placenta and induce arrhythmias in fetal cardiomyocytes, as well as disrupt uteroplacental microcirculation, thereby increasing the risk of fetal hypoxia [4]. Consequently, ICP has been clinically linked to fetal distress, meconium-stained amniotic fluid, spontaneous preterm birth, and even intrauterine fetal demise [5]. In recent years, fetal pulmonary artery Doppler parameters—particularly pulmonary artery acceleration time (PAAT) and ejection time (PAET) have been increasingly used to assess fetal pulmonary vascular resistance and maturation-related hemodynamic changes. However, the effects of ICP on fetal pulmonary circulation remain incompletely understood, and studies examining the relationship between these Doppler parameters, disease severity, and neonatal outcomes are limited [3,6,7].

The aspartate aminotransferase-to-platelet ratio index (APRI) is a reliable, non-invasive biochemical marker used to assess hepatic dysfunction. Although APRI has been primarily applied in chronic liver diseases, its association with elevated liver enzymes in ICP and its potential to reflect biochemical disease severity have recently attracted interest in the obstetric literature [8]. Nevertheless, data evaluating the relationship between APRI and fetal hemodynamic markers particularly pulmonary artery Doppler indices—are scarce [3,8,9]. The present study aims to contribute to the literature by evaluating the fetal pulmonary artery acceleration-to-ejection time ratio (PATET) in pregnancies complicated by ICP and by elucidating its relationship with the clinically relevant APRI score.

## 2. Materials and Methods

### 2.1. Study Design and Population

This prospective, observational, and descriptive study was conducted at the perinatology clinic of a tertiary referral center between 1 February 2025 and 30 June 2025. The study aimed to compare clinical, laboratory, and fetal Doppler parameters between pregnant women diagnosed with intrahepatic cholestasis of pregnancy (ICP) and healthy pregnant women matched for maternal age and gestational age. The study was designed and reported in accordance with the Strengthening the Reporting of Observational Studies in Epidemiology (STROBE) guidelines.

### 2.2. Ethical Approval and Informed Consent

Ethical approval was obtained from the institutional ethics committee of a tertiary referral center prior to study initiation (approval no: 2024/219; approval date: 20 November 2024). All participants were provided with detailed information regarding the study objectives, procedures, and data confidentiality. Written informed consent was obtained from all participants on a voluntary basis. The study was conducted in accordance with the principles of the Declaration of Helsinki (1975, revised in 2013).

### 2.3. Participants and Inclusion Criteria

A total of 128 pregnant women with singleton pregnancies were included in the study. Of these, 64 were diagnosed with intrahepatic cholestasis of pregnancy based on the presence of pruritus during pregnancy accompanied by fasting serum total bile acid levels ≥ 10 µmol/L and a compatible clinical presentation. The remaining 64 participants consisted of healthy pregnant women with no maternal or fetal pathology. Pregnant women presenting with pruritus and elevated liver function tests but normal serum total bile acid levels were not included, as they did not meet the diagnostic criteria for ICP [10].

All participants were between 28 and 40 weeks of gestation. Exclusion criteria included preeclampsia, HELLP syndrome, gestational diabetes mellitus, chronic liver disease, multiple pregnancy, fetal structural anomalies, maternal cardiac disease, and the use of medications known to cause cholestasis.

### 2.4. Definitions and Measurement Parameters

The severity of intrahepatic cholestasis was classified according to fasting serum total bile acid levels into three categories: mild–moderate (10–39 µmol/L), severe (≥40 µmol/L), and very severe (≥100 µmol/L). The AST-to-Platelet Ratio Index (APRI) was calculated using the internationally accepted standard formula, with the upper limit of normal for AST defined according to the laboratory reference range.

Doppler parameters evaluated in this study included pulmonary artery acceleration time (PAAT), pulmonary artery ejection time (PAET), the PAAT/PAET ratio (PATET), umbilical artery pulsatility index (UAPI), umbilical artery resistance index (UARI), middle cerebral artery pulsatility index (MCAPI), and main pulmonary artery Doppler parameters, including MPA pulsatility index (MPA PI), MPA resistance index (MPA RI), MPA systolic/diastolic ratio (MPA S/D), and MPA peak systolic velocity (MPA PSV).

Neonatal outcomes were recorded by an independent neonatologist. The presence of at least one of the following—meconium-stained amniotic fluid, fetal distress, 1 min Apgar score < 7, or need for neonatal intensive care unit (NICU) admission—was defined as a composite adverse neonatal outcome.

### 2.5. Ultrasonography and Doppler Assessments

Fetal Doppler examinations were performed by a single experienced perinatologist using a GE Voluson E10 ultrasound system. Measurements were obtained during fetal apnea, when the fetal heart rate ranged between 120 and 160 beats per minute and fetal breathing movements were minimal. Three consecutive measurements were obtained for each parameter, and the mean value was used for analysis. In the ICP group, Doppler assessments were performed at the time of ICP diagnosis confirmation, concurrent with or within seven days of the diagnostic fasting bile acid measurement, as part of the standardized clinical workup. This design ensured that each Doppler examination captured the hemodynamic state at the point of biochemical diagnosis; however, it represents a single time-point assessment and may not reflect the dynamic evolution of pulmonary vascular hemodynamics over the full course of ICP. To minimize observer bias, the perinatologist performing the fetal Doppler assessments was blinded to the maternal biochemical results, including total fasting bile acid concentrations, AST/ALT values, and the calculated APRI score, at the time of measurement. Biochemical data were retrieved from the laboratory database only after the corresponding Doppler measurements had been completed and stored. Because the diagnosis of ICP was clinically apparent (e.g., maternal pruritus, prior referral information), full operator blinding to group allocation was not feasible; this is acknowledged in the limitation paragraph in the discussion.

**Figure 1:**
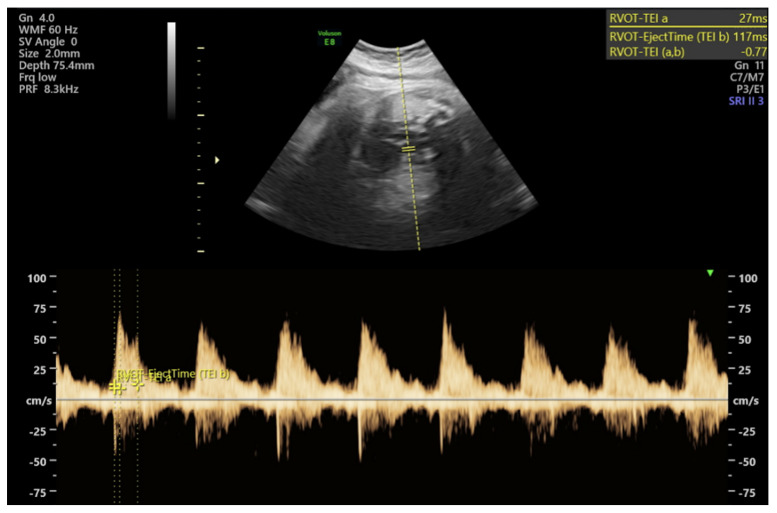
Fetal main pulmonary artery Doppler measurements.

Pulmonary artery Doppler assessment was performed by visualizing the main pulmonary artery in a transverse fetal thoracic section, with the sample volume placed at the level of the pulmonary valve (Figure 1). Umbilical artery Doppler measurements were obtained from a free-floating loop of the umbilical cord, while middle cerebral artery Doppler measurements were performed at the level of the circle of Willis from the proximal segment with optimal flow visualization.

Intraobserver reliability was assessed at the beginning of the study. The intraclass correlation coefficients (ICC) for repeated measurements were 0.92 for PAAT, 0.90 for PAET, and 0.91 for PATET (all *p* < 0.001).

### 2.6. Assessment of Neonatal Outcomes

Neonatal outcomes were assessed immediately after delivery by a neonatology specialist. Apgar scores were calculated at 1 and 5 min using the standard method. The need for neonatal intensive care unit (NICU) admission was determined based on clinical evaluation regarding requirement for respiratory support and/or other neonatal interventions. The presence of meconium was recorded by the delivery team during labor.

### 2.7. Statistical Analysis

All statistical analyses were performed using SPSS software (SPSS v26.0). The normality of continuous variables was assessed using the Kolmogorov–Smirnov test. Normally distributed variables are presented as mean ± standard deviation, and between-group comparisons were performed using Student’s *t* test. For variables not following a normal distribution, the Kruskal–Wallis test was applied. Categorical variables are presented as numbers and percentages, and comparisons were conducted using Pearson’s chi-square test. Associations between PATET and APRI and fasting bile acids (FBA) were evaluated using Spearman correlation analysis. Statistical significance was set at *p* < 0.05. There were no missing data; therefore, all analyses were conducted using the complete dataset. Among baseline variables, BMI was the only one showing a statistically significant between-group imbalance (*p* = 0.049) and was therefore prespecified as the principal covariate; all key between-group and severity-stratified comparisons were adjusted for BMI using ANCOVA (or rank-based ANCOVA) for continuous outcomes and binary logistic regression for categorical outcomes. Other potential confounders—including gestational age at Doppler assessment, mode of delivery, and exposure to ursodeoxycholic acid (UDCA) or other ICP-directed pharmacotherapies—were not entered into the adjusted models because of sample-size constraints and the partly downstream nature of these variables relative to ICP; this point is explicitly acknowledged in the Limitations section.

A post hoc power analysis was conducted using G*Power software (v3.1). For the primary between-group comparison (n = 64 per group), the study achieved 80% power to detect a medium effect size (Cohen’s d = 0.50) at a two-sided alpha of 0.05. For the severity subgroup analyses, however, the markedly unequal and small group sizes—particularly the very severe ICP subgroup (n = 9)—substantially limited statistical power. Post hoc calculations estimated approximately 56% power to detect a large effect size (f = 0.40; η^2^ = 0.14) in the three-group comparison, falling well below the conventional 80% threshold. Accordingly, non-significant findings in the severity-stratified analyses must be interpreted with caution, and the possibility of a type II error (false-negative result) cannot be excluded.

## 3. Results

A total of 128 participants were included in the study: 64 pregnant women with ICP and 64 healthy controls. BMI was significantly lower in the ICP group than in controls (29.17 ± 3.30 vs. 30.34 ± 3.36 kg/m^2^; *p* = 0.049). Given this baseline difference, all subsequent between-group comparisons were adjusted for BMI using ANCOVA (parametric or rank-based) for continuous variables and logistic regression for categorical variables.

In the ICP group, the number of living children was significantly lower compared with controls (*p* < 0.001). Women in the ICP group delivered at an earlier gestational age (*p* < 0.001). Neonatal birth weight was numerically lower in the ICP group; however, after BMI adjustment, this difference did not reach statistical significance (unadjusted *p* = 0.039; BMI-adjusted *p* = 0.064). The 5 min Apgar score was significantly lower in the ICP group even after BMI adjustment (*p* = 0.003). The 1 min Apgar < 7 rate did not differ between groups (ICP 6.3% vs. Control 4.7%; Fisher’s exact *p* = 1.000). The cesarean delivery rate was higher in the ICP group but remained borderline after adjustment (*p* = 0.058). No significant between-group differences were observed in meconium-stained amniotic fluid, fetal distress, NICU admission, or the composite adverse neonatal outcome after BMI adjustment (all *p* > 0.05). Maternal characteristics and perinatal outcomes are summarized in Table 1.

Doppler evaluation demonstrated that PAAT, PAET, PATET, and UAPI values were significantly higher in the ICP group after BMI adjustment (all *p* < 0.05). MCAPI did not differ significantly between groups (*p* = 0.335). Among liver function tests, AST, ALT, and APRI values were markedly elevated in the ICP group (all *p* < 0.001). Platelet count was numerically lower in the ICP group; however, after BMI adjustment, this difference lost statistical significance (unadjusted *p* = 0.039; BMI-adjusted *p* = 0.072). Comparisons of laboratory and Doppler parameters are presented in Table 2.

When stratified by ICP severity, no significant differences were observed among groups in pulmonary artery Doppler parameters (PAAT, PAET, PATET), umbilical and middle cerebral artery indices (UAPI, UARI, MCAPI), or main pulmonary artery measurements (MPA PI, MPA RI, MPA S/D, and MPA PSV) after BMI adjustment (all *p* > 0.05). Similarly, liver function tests (AST and ALT) and platelet count did not differ significantly across severity categories. APRI showed a borderline trend (Kruskal–Wallis *p* = 0.068; BMI-adjusted *p* = 0.065), with mean values tending to increase with increasing severity without reaching statistical significance. Comparisons according to ICP severity are shown in Table 3.

PATET showed positive but non-significant associations with both APRI (Spearman r = 0.114, *p* = 0.368; BMI-adjusted partial r = 0.114, *p* = 0.375) and FBA (Spearman r = 0.198, *p* = 0.116; BMI-adjusted partial r = 0.209, *p* = 0.099). In contrast, APRI demonstrated a weak but statistically significant positive correlation with FBA, which was preserved after BMI adjustment (Spearman r = 0.308, *p* = 0.013; BMI-adjusted partial r = 0.310, *p* = 0.013). Correlation analyses within the ICP group are summarized in Table 4.

When ICP cases were compared according to three adverse neonatal markers, neither PATET nor APRI differed significantly after BMI adjustment (all *p* > 0.05). Although APRI tended to be numerically higher in neonates with meconium-stained amniotic fluid, fetal distress, or NICU admission, none of these differences reached statistical significance. Distributions of PATET and APRI according to adverse neonatal markers within the ICP group are shown in Table 5.

## 4. Discussion

In this study, fetal pulmonary hemodynamics and maternal liver function-related biomarkers were comprehensively evaluated in pregnancies complicated by intrahepatic cholestasis of pregnancy (ICP). The main findings indicate that PAAT, PAET, and the PATET ratio were significantly higher in the ICP group than in healthy controls. Umbilical artery Doppler indices were also significantly increased in the ICP group. Among biochemical markers, AST, ALT, and APRI values were markedly elevated in ICP. Nevertheless, no significant association was observed between PATET and APRI or fasting bile acid levels. In addition, subgroup analyses according to cholestasis severity revealed no statistically significant differences in Doppler parameters or APRI. Clinically, earlier delivery, lower birth weight, and lower 5 min Apgar scores were observed in the ICP group; however, no significant between-group difference was found in composite adverse neonatal outcomes. Collectively, these results suggest that ICP affects both fetal pulmonary circulation and maternal hepatobiliary parameters, yet these physiological processes may behave independently. Moreover, although adverse neonatal markers and pulmonary artery Doppler indices were numerically higher in ICP, no pathological relationship between these changes was demonstrated.

Similarly, Niculae et al. published a comprehensive review reporting that ICP increases the risks of neonatal morbidity, lower Apgar scores, and meconium-stained amniotic fluid [11]. The lower 5 min Apgar scores observed in our ICP group are consistent with these findings. However, the absence of statistically significant differences in meconium staining and fetal distress aligns with studies suggesting that neonatal risks may be more limited, particularly in mild-to-moderate ICP [11,12,13]. Although the cesarean delivery rate was higher in the ICP group, it did not reach statistical significance; this partially overlaps with meta-analytic evidence indicating that ICP may increase cesarean rates [14], while differences in sample distribution and clinical practice patterns may have influenced our result. Overall, our findings are in agreement with the existing body of evidence identifying ICP as an important condition affecting preterm delivery and fetal well-being.

Evidence from non-ICP populations suggests that PATET may serve as a sensitive marker of fetal pulmonary function. For instance, Moety et al. reported that the fetal pulmonary artery AT/ET ratio has meaningful accuracy in predicting neonatal respiratory distress syndrome [15]. Likewise, Yakıştıran et al. demonstrated significant differences in PAAT, PAET, and PATET between pregnancies complicated by ICP and controls, supporting the concept that PATET may reflect ICP-related alterations in fetal pulmonary circulation [3]. In line with these reports, our results indicate an increase in pulmonary artery Doppler indices in ICP, suggesting altered fetal pulmonary hemodynamics. Importantly, an elevation in PATET—reflecting relative prolongation of the pulmonary artery acceleration phase in relation to total ejection time—physiologically indicates lower rather than higher pulmonary vascular resistance (PVR), consistent with accelerated fetal lung maturation or a compensatory hemodynamic adaptation under ICP-related physiological perturbations. However, we did not identify a significant association between Doppler indices and neonatal morbidity measures, suggesting that elevated pulmonary Doppler parameters may not directly translate into clinically detectable short-term neonatal compromise in our cohort.

The lack of significant differences in Doppler indices across cholestasis severity categories is also consistent with reports indicating that fetal hemodynamic effects may not be linearly dependent on total bile acid levels. Serra et al. noted that routine fetoplacental Doppler parameters often remain within normal limits in ICP and that hemodynamic changes may not necessarily parallel biochemical severity [16]. Furthermore, a systematic review and meta-analysis by Zhan et al. emphasized the heterogeneity of fetal cardiac and hemodynamic responses in ICP and highlighted the potential role of individual and placental adaptations [17]. This underscores the complexity of ICP-related effects on fetoplacental circulation. Taken together, our findings support the interpretation that ICP may influence fetal pulmonary artery flow patterns and fetoplacental circulation, while the magnitude of this effect may vary independently of biochemical disease severity.

In the present study, APRI was significantly higher in the ICP group and demonstrated a weak-to-moderate positive correlation with bile acid levels, in line with emerging data suggesting that APRI may serve as a diagnostic and/or severity-related biomarker in ICP. Two recent studies reported significantly increased APRI in ICP compared with controls and suggested good discriminatory performance for ICP diagnosis, with an area under the curve (AUC) up to approximately 0.84 [8,18]. Similarly, in a series of 101 ICP cases, APRI was higher in severe ICP, and a significant positive association was reported between APRI and bile acids (r ≈ 0.45) [19]. Our observation of a positive APRI–fasting bile acid correlation is consistent with these findings.

Conversely, although AST and ALT are commonly used in the diagnostic evaluation of ICP, strong evidence supports total bile acids as the primary biomarker for predicting adverse perinatal outcomes. In an individual patient data meta-analysis including 5269 ICP cases, bile acids ≥ 100 µmol/L were strongly associated with stillbirth risk, whereas ALT showed no meaningful association with stillbirth (ROC AUC ≈ 0.46) [20]. Therefore, APRI may provide limited added value as an adjunct reflecting maternal hepatocellular stress; however, it should not be considered a substitute for bile acids in risk stratification [20,21].

The absence of significant differences in Doppler parameters (PAAT, PAET, PATET), umbilical and cerebral artery indices, or APRI and liver enzymes with increasing cholestasis severity further supports the concept that biochemical severity does not always mirror fetal hemodynamic alterations in ICP. Ovadia et al. demonstrated that fetal risk increases substantially mainly at bile acid levels ≥ 100 µmol/L, while other liver tests have limited predictive value [20]. Similarly, some studies have reported impaired fetal cardiac function in ICP, yet emphasized that these alterations do not consistently correlate linearly with serum bile acids [22,23,24]. Nevertheless, evidence showing significant associations with total bile acids also exists [25]. In our cohort, the small number of very severe ICP cases, fluctuations in bile acid levels during pregnancy, and inter-individual differences in placental adaptation may explain the lack of statistically significant differences across severity categories.

The observed increase in PAAT, PAET, and PATET in ICP suggests that cholestasis may be associated with hemodynamic alterations in fetal pulmonary circulation. Experimental and clinical observations have previously indicated that bile acids can cross the placental barrier and may affect fetal cardiomyocytes and pulmonary vascular structures. This provides a biologically plausible basis for the Doppler flow pattern changes detected in our study. Mechanistically, several pathways may link elevated maternal bile acids to altered fetal pulmonary artery hemodynamics. Hydrophobic bile acids—particularly taurocholic and glycocholic acid—readily cross the placenta and accumulate in the fetal compartment, where in vitro and ex vivo studies have demonstrated vasoactive effects on placental and chorionic vessels and impairment of endothelial nitric oxide-mediated relaxation. In the fetal pulmonary circulation, bile acid exposure may interact with surfactant homeostasis and pulmonary epithelial maturation, potentially promoting maturation-related reductions in pulmonary vascular resistance (PVR) rather than increases. Bile acids also exert direct electrophysiological and contractile effects on fetal cardiomyocytes, potentially altering right ventricular contractility and ejection dynamics. Critically, an elevation in PATET reflects a relative prolongation of acceleration time in relation to total ejection time, which is physiologically consistent with lower—rather than higher—PVR. This pattern may indicate bile acid-driven acceleration of fetal lung maturation, endothelial vasodilatory responses, or altered right ventricular ejection dynamics secondary to cardiomyocyte effects—all resulting in the observed upward shift in the PATET ratio. The persistence of these Doppler changes despite the absence of a significant correlation with biochemical severity supports the view that fetal pulmonary effects in ICP are likely mediated through individual susceptibility, placental adaptation, and time-dependent bile acid exposure rather than through a single absolute threshold of maternal bile acid concentration.

The lack of a significant association between APRI and Doppler parameters despite markedly elevated APRI values suggests that maternal hepatocellular injury in ICP does not necessarily progress in parallel with fetal hemodynamic changes. Consistent with the meta-analysis by Ovadia et al., fetal risk appears to be primarily driven by bile acid concentrations, while AST/ALT and related markers have limited ability to predict perinatal risk [20]. Thus, our findings suggest that ICP may influence fetal pulmonary circulation, but this effect may not closely track biochemical severity. Placental adaptation, individual fetal susceptibility, and temporal variability in bile acid concentrations may represent plausible pathophysiological explanations for this dissociation.

It should be emphasized that, although our findings suggest a possible complementary role for PATET in the surveillance of ICP pregnancies, we did not perform a formal predictive analysis (e.g., ROC curve construction with calculation of sensitivity, specificity, optimal cut-off values, or the area under the curve) for adverse perinatal outcomes such as meconium-stained amniotic fluid, fetal distress, low Apgar score, or NICU admission. The relatively low frequency of severe adverse events in our cohort, together with the small numbers in the severe and very severe ICP subgroups, precluded a stable estimation of the predictive performance of PATET or APRI. The non-significant numerical trends observed in Table 5—with both PATET and APRI being slightly higher in pregnancies complicated by adverse neonatal markers—suggest that an exploratory predictive role cannot be excluded but should be regarded as hypothesis-generating only. Adequately powered, multicenter studies with prospective registration of perinatal outcomes are required to formally test whether PATET, alone or in combination with APRI and serial bile acid measurements, can be incorporated into a clinically useful risk-stratification or prediction model for ICP-related adverse perinatal outcomes.

Regarding the intended clinical role of PATET in the management of ICP, the present findings support its characterization as a hemodynamic monitoring variable rather than a standalone screening test or prognostic indicator. Bile acid levels remain the gold standard for predicting adverse neonatal outcomes in ICP [20], and PATET should not be considered a substitute for biochemical risk stratification. Instead, its potential clinical utility may lie in serial hemodynamic monitoring—tracking changes in fetal pulmonary vascular adaptation throughout the course of ICP management as a complement to bile acid measurements. This may be particularly informative in cases with borderline bile acid levels or when clinical decisions regarding delivery timing are under consideration. Whether the incorporation of PATET into a multiparameter surveillance algorithm meaningfully improves outcomes beyond standard bile acid monitoring requires validation in prospective, adequately powered studies.

A key strength of this study is the concurrent assessment of fetal pulmonary artery hemodynamics and maternal liver function-related biomarkers, particularly APRI, in pregnancies with ICP. As studies evaluating PATET and APRI simultaneously are scarce, this work provides a novel contribution. Measurements were obtained at comparable gestational ages, Doppler parameters were evaluated using a standardized protocol by a single experienced team, and baseline maternal characteristics were broadly similar between groups, supporting internal validity. In addition, all biochemical tests were analyzed in the same laboratory using the same methodology, thereby reducing systematic measurement variability.

Several limitations should be considered. First, the sample size, particularly within the severe and very severe ICP subgroups, was limited, which may have reduced statistical power to detect between-group differences. Because bile acid levels can fluctuate over time, severity classification based on a single measurement may not fully reflect true biological severity. Moreover, Doppler assessments were performed in later gestational weeks, limiting evaluation of earlier hemodynamic changes. As this was a single-center study, generalizability may be limited. Finally, despite the prospective design, long-term neonatal outcomes were not assessed, which restricts comprehensive evaluation of fetal effects. The relatively small numbers in the severe (n = 19) and very severe (n = 9) ICP subgroups markedly reduce the reliability of conclusions regarding severity-dependent effects on PATET, APRI, and the other Doppler indices; accordingly, the absence of statistically significant differences across severity strata should be interpreted as inconclusive rather than as evidence of no effect, and a type II error cannot be ruled out. As detailed in the Statistical Analysis section, post hoc power calculations confirmed that the three-group severity comparison had only approximately 56% power to detect a large effect size (f = 0.40), substantially below the conventional 80% threshold; accordingly, the possibility of type II error is explicitly acknowledged for all severity-stratified comparisons. Severity classification was based on a single fasting bile acid measurement and therefore did not capture the dynamic intra-individual fluctuations of bile acid concentrations that occur during pregnancy; serial sampling, ideally synchronized with Doppler assessments, would have provided a more robust phenotype and is recommended in future studies. Additionally, both Doppler and biochemical evaluations were performed as single time-point assessments at the time of ICP diagnosis; ICP is a dynamic condition in which bile acid levels and fetoplacental hemodynamics may fluctuate substantially over the course of pregnancy. The interval between Doppler assessment and delivery was variable and not fully standardized, and measurements obtained at different disease stages may reflect different hemodynamic states. Serial synchronized Doppler and biochemical assessments—performed at diagnosis, during treatment escalation, and close to term—would provide a more comprehensive characterization of the temporal relationship between maternal cholestasis and fetal pulmonary hemodynamics. Furthermore, although BMI was the only baseline variable showing a clinically meaningful imbalance and was therefore prespecified as the principal covariate, other potentially relevant confounders—including gestational age at Doppler assessment, mode of delivery, and exposure to ursodeoxycholic acid (UDCA) or other ICP-directed pharmacotherapy—were not formally included in the multivariable models because of sample-size constraints and because some of these variables (e.g., delivery mode) are partly downstream of ICP itself. Residual confounding from these variables, especially with respect to neonatal outcomes, cannot therefore be excluded. We did not perform a formal predictive (ROC-based) analysis of PATET or APRI for adverse perinatal outcomes; consequently, the proposal that PATET may serve as a complementary surveillance tool should be regarded as hypothesis-generating and requires validation in adequately powered prospective studies. With respect to operator-related bias, all Doppler measurements were performed by a single experienced perinatologist who was blinded to the maternal biochemical results; however, full blinding to ICP status was not feasible because of the clinical apparent presentation, and the single-operator, single-center design inevitably limits external generalizability.

Our findings suggest that ICP may affect fetal pulmonary circulation and that pulmonary artery Doppler parameters (PAAT, PAET, and PATET) may provide potentially useful adjunctive information during clinical assessment. The lack of association between APRI and fetal hemodynamic parameters despite elevated APRI values highlights a possible dissociation between maternal hepatocellular stress and fetal circulatory adaptation in ICP.

From a clinical perspective, the evaluation of fetal well-being in ICP may benefit from integrating fetal hemodynamic findings in addition to biochemical markers. However, both our subgroup findings and the existing literature indicate that the relationship between cholestasis severity and Doppler indices is not consistent. Therefore, larger prospective studies are required before Doppler parameters can be routinely incorporated into surveillance algorithms.

Future research should incorporate serial bile acid measurements with synchronized Doppler assessments to better characterize dynamic effects on fetoplacental circulation. Multicenter studies would also help validate the clinical utility and reproducibility of parameters such as PATET across different populations.

In conclusion, this study is among the few to jointly evaluate fetal pulmonary artery hemodynamics and maternal liver function-related biomarkers, particularly APRI, in pregnancies complicated by ICP. We demonstrated significantly increased PAAT, PAET, and PATET in the ICP group, whereas APRI did not correlate with fetal hemodynamic indicators. The lack of a clear change in Doppler parameters with increasing cholestasis severity suggests that fetal circulatory effects may not be solely determined by biochemical severity. Overall, fetal hemodynamic changes and maternal liver biochemistry may follow independent courses in ICP, and this area warrants further investigation through larger, serial-measurement studies.

## Figures and Tables

**Table 1:** Comparison of maternal characteristics and perinatal outcomes between the ICP group and healthy controls (BMI-adjusted).

Variable	ICP (*n* = 64)	Control (*n* = 64)	*p* (Unadj)	*p* (BMI-Adj)	Test
Maternal age (years)	28.00 ± 4.27	29.11 ± 5.33	0.196	0.326	t/ANCOVA
Gravidity	1.89 ± 1.18	2.11 ± 1.13	0.150	0.110	MWU/R-ANC
Parity	0.72 ± 0.84	0.64 ± 0.74	0.735	0.929	MWU/R-ANC
Miscarriage	0.23 ± 0.61	0.19 ± 0.53	0.766	0.679	MWU/R-ANC
Living children	0.42 ± 0.56	0.88 ± 0.85	0.001	<0.001	MWU/R-ANC
BMI (kg/m^2^)	29.17 ± 3.30	30.34 ± 3.36	0.049	—	*t*-test
GA at delivery (wk)	36.64 ± 0.74	37.95 ± 1.42	<0.001	<0.001	MWU/R-ANC
Birth weight (g)	3048.05 ± 386.66	3187.02 ± 367.86	0.039	0.064	t/ANCOVA
1 min Apgar	7.66 ± 0.60	7.78 ± 0.58	0.103	0.202	MWU/R-ANC
5 min Apgar	8.73 ± 0.45	8.94 ± 0.24	0.002	0.003	MWU/R-ANC
1 min Apgar <7, n (%)	4 (6.3%)	3 (4.7%)	1.000	—	Fisher
Cesarean, n (%)	50 (78.1%)	40 (62.5%)	0.081	0.058	LogReg
Meconium, n (%)	9 (14.1%)	4 (6.2%)	0.241	0.214	LogReg
Fetal distress, n (%)	9 (14.1%)	8 (12.5%)	1.000	0.954	LogReg
NICU admission, n (%)	15 (23.4%)	14 (21.9%)	1.000	0.844	LogReg
Composite adverse, n (%)	16 (25.0%)	15 (23.4%)	—	0.856	LogReg

Data are presented as mean ± SD or n (%). Unadjusted p: Student’s *t*-test or Mann–Whitney U for continuous variables; Fisher’s exact or chi-square for categorical variables. BMI-adjusted *p*: ANCOVA or rank-based ANCOVA for continuous variables; logistic regression for categorical variables. MWU/R-ANC = Mann–Whitney U/Rank-based ANCOVA.

**Table 2:** Comparison of laboratory and Doppler parameters between the ICP group and healthy controls (BMI-adjusted).

Variable	ICP (*n* = 64)	Control (*n* = 64)	*p* (Unadj)	*p* (BMI-Adj)	Test
PAAT (s)	0.0545 ± 0.0518	0.0450 ± 0.0535	<0.001	<0.001	MWU/R-ANC
PAET (s)	0.2059 ± 0.0361	0.1951 ± 0.0035	<0.001	<0.001	MWU/R-ANC
PATET ratio	0.2231 ± 0.0484	0.1809 ± 0.0074	<0.001	<0.001	MWU/R-ANC
UAPI	0.8879 ± 0.1616	0.8234 ± 0.1032	0.016	0.014	MWU/R-ANC
MCAPI	1.5639 ± 0.0839	1.5459 ± 0.0935	0.255	0.335	t/ANCOVA
AST (U/L)	110.42 ± 86.80	17.95 ± 5.77	<0.001	<0.001	MWU/R-ANC
ALT (U/L)	178.47 ± 140.06	14.11 ± 3.78	<0.001	<0.001	MWU/R-ANC
PLT (/µL)	228,453 ± 60,379	253,766 ± 76,206	0.039	0.072	t/ANCOVA
APRI	1.2053 ± 0.8186	0.2681 ± 0.0721	<0.001	<0.001	MWU/R-ANC

Data are presented as mean ± SD. Fasting bile acids (FBA) were measured only in the ICP group. MWU/R-ANC = Mann–Whitney U/Rank-based ANCOVA.

**Table 3:** Comparison of Doppler and laboratory parameters according to ICP severity (BMI-adjusted).

Parameter	Mild-Mod (*n* = 36)	Severe (*n* = 19)	Very Severe (*n* = 9)	*p* (KW)	*p* (adj)
PAAT	0.049 ± 0.003	0.069 ± 0.095	0.048 ± 0.004	0.412	0.440
PAET	0.213 ± 0.024	0.194 ± 0.050	0.202 ± 0.037	0.395	0.373
PATET	0.218 ± 0.052	0.229 ± 0.045	0.230 ± 0.041	0.405	0.440
UAPI	0.898 ± 0.173	0.852 ± 0.152	0.922 ± 0.131	0.478	0.519
MCAPI	1.553 ± 0.072	1.576 ± 0.105	1.580 ± 0.082	0.329	0.360
AST (U/L)	88.14 ± 51.59	135.26 ± 116.53	147.11 ± 109.17	0.330	0.306
ALT (U/L)	154.08 ± 110.95	193.84 ± 185.13	243.56 ± 124.57	0.187	0.188
PLT (×10^3^)	236.5 ± 54.2	216.0 ± 71.0	222.7 ± 61.5	0.403	0.434
APRI	0.977 ± 0.607	1.497 ± 0.940	1.505 ± 1.067	0.068	0.065
UARI	0.599 ± 0.039	0.592 ± 0.018	0.586 ± 0.007	0.875	0.881
MPA PI	2.168 ± 0.001	2.167 ± 0.004	2.168 ± 0.001	0.151	0.163
MPA RI	0.857 ± 0.002	0.858 ± 0.002	0.857 ± 0.001	0.527	0.539
MPA S/D	7.866 ± 1.333	8.088 ± 0.001	7.199 ± 2.667	0.572	0.557
MPA PSV	83.74 ± 0.33	83.71 ± 0.39	75.10 ± 25.04	0.126	0.123

Severity groups based on fasting serum total bile acid levels: 10–39 µmol/L (mild–moderate), ≥40 µmol/L (severe), ≥100 µmol/L (very severe). Data: mean ± SD. Kruskal–Wallis test (unadjusted); rank-based ANCOVA with BMI covariate (adjusted).

**Table 4:** Correlation analysis among PATET, APRI, and fasting bile acids (FBAs) in the ICP group (with BMI-adjusted partial correlations).

Variable Pair	r (Spearman)	*p*	Significance	r (Partial)	*p* (Adj)	Significance
PATET–APRI	0.114	0.368	No	0.114	0.375	No
PATET–FBA	0.198	0.116	No	0.209	0.099	No
APRI–FBA	0.308	0.013	Yes	0.310	0.013	Yes

Bivariate correlations: Spearman’s rho. Partial correlations: rank-based partial correlation controlling for BMI.

**Table 5:** Comparison of PATET and APRI according to adverse neonatal markers in the ICP group (BMI-adjusted).

Variable	Cat	n	PATET	APRI	*p* (PATET)	*p* (APRI)	Adj
Meconium	No	55	0.225 ± 0.050	1.139 ± 0.757			
	Yes	9	0.211 ± 0.038	1.608 ± 1.093	0.678	0.395	NS
Fetal distress	No	55	0.226 ± 0.050	1.176 ± 0.816			
	Yes	9	0.207 ± 0.036	1.387 ± 0.862	0.275	0.602	NS
NICU	No	49	0.222 ± 0.047	1.077 ± 0.638			
	Yes	15	0.227 ± 0.054	1.625 ± 1.172	0.692	0.222	NS

Data are presented as mean ± SD. Analyses within the ICP group only (n = 64). Unadjusted: Mann–Whitney U test. Adjusted: rank-based ANCOVA controlling for BMI. NS = not significant after BMI adjustment.

## Data Availability

Data are not publicly available due to ethical restrictions. Further inquiries can be directed to the corresponding author.

## Abbreviations

The following abbreviations are used in this manuscript:

ICP	Intrahepatic Cholestasis of Pregnancy
PAAT	Pulmonary Artery Acceleration Time
PAET	Pulmonary Artery Ejection Time
PATET	Pulmonary Artery Acceleration Time to Ejection Time Ratio
APRI	Aspartate Aminotransferase-to-Platelet Ratio Index
UAPI	Umbilical Artery Pulsatility Index
MCAPI	Middle Cerebral Artery Pulsatility Index
AST	Aspartate Aminotransferase
ALT	Alanine Aminotransferase
FBA	Fasting Bile Acids
UARI	Umbilical Artery Resistance Index
MPA	Main Pulmonary Artery
MPA PI	Main Pulmonary Artery Pulsatility Index
MPA RI	Main Pulmonary Artery Resistance Index
MPA S/D	Main Pulmonary Artery Systolic/Diastolic Ratio
MPA PSV	Main Pulmonary Artery Peak Systolic Velocity
NICU	Neonatal Intensive Care Unit
BMI	Body Mass Index
ANCOVA	Analysis of Covariance
MWU	Mann–Whitney U Test
R-ANC	Rank-based Analysis of Covariance
SPSS	Statistical Package for the Social Sciences
ICC	Intraclass Correlation Coefficient
